# Autism stigma, knowledge, attitude, and quality of life predictors among autistic children’s caregivers

**DOI:** 10.3389/fpsyg.2026.1762237

**Published:** 2026-05-21

**Authors:** Hend A. Alnajjar, Neama Y. Hantira, Juna Aljadani, Marwah Almoteri, Munira Arous, Maha Alamari, Majd Albogami, Ghadi Abu Alola, Reyouf Alzahrani, Razan Almotiri, Layla Wasli, Deemah Bajbair

**Affiliations:** 1 College of Nursing, King Saud bin Abdulaziz University for Health Sciences, Jeddah, Saudi Arabia; 2 King Abdullah International Medical Research Center (KAIMRC), Jeddah, Saudi Arabia; 3 Ministry of the National Guard—Health Affairs, Jeddah, Saudi Arabia; 4 Faculty of Nursing, Alexandria University, Alexandria, Egypt

**Keywords:** attitude, autism spectrum disorder, caregivers, knowledge, predictors, quality of life, self-stigma

## Abstract

**Background:**

Autism spectrum disorder (ASD) is a complex neurodevelopmental condition and a common challenge in early childhood, affecting approximately one in every hundred children globally. This study assessed the knowledge, stigma, attitudes, and quality of life (QoL) of caregivers of autistic children in Jeddah, Saudi Arabia.

**Methods:**

A cross-sectional descriptive design was conducted from January to March 2024 at Bagedo Dr. Erfan Hospital and Jeddah Autism Center for Day Care, involving 210 caregivers selected through simple random sampling. Data collection tools included assessments of caregivers’ sociodemographic and child characteristics, stigma, attitudes, and QoL.

**Results:**

Over half of the caregivers had poor knowledge, and two-thirds exhibited negative attitudes toward autism, with a moderate level of overall stigma among caregivers. Additionally, more than three-quarters reported inadequate QoL. Multiple linear regression analysis revealed a statistically significant model (*F* = 7.233, *p* < 0.001), explaining 24.7% of the variance in QoL. Caregiver stigma was the strongest negative predictor (*β* = −0.401, *p* < 0.001), while higher caregiver knowledge significantly improved QoL (*β* = 0.253, *p* < 0.001). Having a female child was associated with lower QoL, whereas higher birth order predicted better QoL.

**Conclusion:**

Caregivers of autistic children demonstrated poor QoL, significantly associated with stigma, knowledge, and selected child characteristics. Reducing stigma and enhancing caregiver knowledge are critical to improving QoL.

**Recommendations:**

Public awareness initiatives are essential to reduce stigma and improve the well-being of caregivers of autistic children.

## Introduction

Autism spectrum disorder (ASD) is one of the fastest-growing neurodevelopmental disorders affecting both brain and overall functioning (Mukhamedshina et al., 2022). It is genetically influenced, often identifiable by the age of 2 years, and is more prevalent among males and preterm children (Anwar et al., 2018). ASD is characterized by persistent challenges in social communication, along with restricted interests and repetitive behaviors (Hodges et al., 2020). Globally, approximately one in every 100 children is diagnosed with autism (Zeidan et al., 2022).

Stigma remains a significant challenge for children with ASD and their families. It is defined as a process in which societal reactions devalue and spoil an individual’s identity, leading to parental humiliation and social marginalization (Goffman, 2022; Ng and Ng, 2022). Parents of children with disabilities, including ASD, are more likely to experience stigmatizing attitudes compared to others (Alshaigi et al., 2020). Therefore, public awareness and education are essential in reducing stigma and fostering positive attitudes toward ASD, as caregivers’ perceptions significantly influence family well-being and the quality of care. In this context, parental adjustment plays a crucial role in managing these challenges (Bakhet, 2017).

Parental adjustment involves managing emotional responses to a child’s condition while balancing daily stressors with available personal and social resources, such as partner support. Effective adjustment promotes emotional stability and enables parents to maintain self-image, preserve family cohesion, and prepare for ongoing uncertainties (Mohammadi et al., 2020). As primary caregivers, parents’ ability to recognize and respond to signs of autism is essential for providing appropriate care. Adequate knowledge reduces the risk of misdiagnosis, facilitates timely intervention, and supports better health outcomes. Such awareness can be enhanced through various information sources, including media platforms (Anwar et al., 2018).

In this regard, social media may contribute to raising public awareness about ASD by providing access to reliable information from credible sources and healthcare professionals (Van de Belt et al., 2013). Previous studies have consistently demonstrated an inverse relationship between caregiving burden and quality of life (QoL) among caregivers of children with ASD (Gabra et al., 2021; Renford et al., 2020; Khan et al., 2020). The demands associated with caring for an autistic child can significantly impact caregivers’ QoL due to the ongoing emotional, social, and practical challenges involved (Al-Jabri et al., 2022).

According to the World Health Organization, quality of life (QoL) is defined as an individual’s perception of their position in life within the context of their culture and value systems, in relation to their goals, expectations, standards, and concerns (World Health Organization, 1996a). This multidimensional concept encompasses physical, psychological, emotional, social, and material well-being (Guerra-Martín et al., 2023). Evidence indicates that caregivers of children with ASD often experience psychosocial difficulties, including stress, depression, anxiety, marital strain, and poor physical health. These unique caregiving demands highlight the importance of examining QoL among this population and understanding the factors that influence it (Padden and James, 2017; Tathgur and Kang, 2021; Losada-Puente et al., 2022). A study conducted in Jeddah by Al-Jabri et al. reported that caregivers of children with ASD had significantly lower QoL compared to caregivers of children without ASD (Al-Jabri et al., 2022). Nurses play a vital role in supporting these families through screening, early detection, diagnosis, referral, education, advocacy, and facilitating access to community resources (Neyoshi, 2018; Jennings and Pauline, 2020; Saad et al., 2020).

The present study aims to assess the levels of knowledge, stigma, attitudes, and quality of life among caregivers of children with autism in Jeddah, Saudi Arabia, and to examine the predictors of these outcomes through regression analysis.

### Significance of the study

Autism spectrum disorder (ASD) is a common neurodevelopmental condition affecting children from early childhood. In Saudi Arabia, reported prevalence rates include 2.618 per 1,000 children in Jeddah, 3.68 per 1,000 in Makkah, and 2.81 per 1,000 in both cities (Sabbagh et al., 2021). ASD places substantial and continuous demands on families, with caregivers facing long-term responsibilities and challenges throughout the caregiving process (Losada-Puente et al., 2022). Improving caregivers’ quality of life requires adequate knowledge about autism, positive attitudes, and effective coping strategies. However, existing evidence suggests the presence of inadequate knowledge and negative attitudes toward autism among the public (Alyami et al., 2022). Nurses are central in addressing caregivers’ needs, supporting emotional coping, and acting as a link between families and healthcare systems. Therefore, examining caregivers’ experiences is essential to inform interventions aimed at improving their quality of life (Losada-Puente et al., 2022).

### Theoretical and conceptual framework

**Figure 1:**
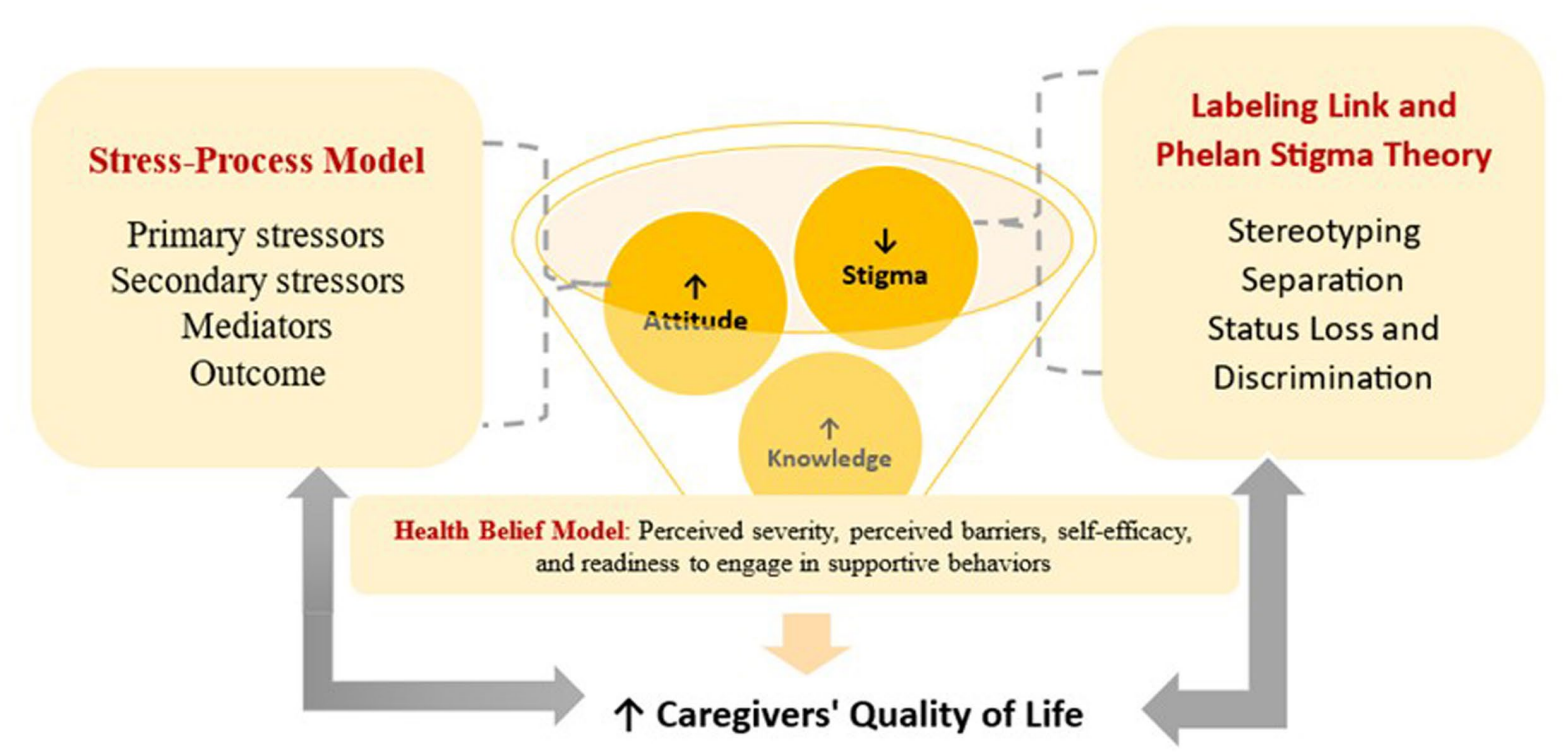
Theoretical and conceptual framework of the study (created by: Author NH).

The present study is guided by an integrated theoretical framework combining Link and Phelan’s Stigma Theory, the Stress-Process Model, and the Health Belief Model (Figure 1). Link and Phelan conceptualize stigma as a process involving labeling, stereotyping, social separation, and discrimination, which collectively shape caregivers’ lived experiences. The Stress-Process Model explains how primary caregiving demands and secondary stressors such as public stigma, self-stigma, and social exclusion contribute to psychological distress and diminished quality of life. Within this framework, knowledge about autism and positive attitudes function as protective and mediating factors that enhance coping and reduce stress. From the perspective of the Health Belief Model, caregivers’ knowledge influences perceived severity, perceived barriers, self-efficacy, and readiness to engage in appropriate caregiving behaviors, ultimately affecting their quality of life. Collectively, these theoretical perspectives suggest that stigma increases caregiver burden and negatively impacts well-being, whereas knowledge and positive attitudes serve as key determinants of improved quality of life among caregivers of children with ASD (Pearlin et al., 1981; Link and Phelan, 2013; Alyafei and Easton-Carr, 2025).

## Methodology

### Study design

A cross-sectional descriptive study design was used.

### Study settings

The study was conducted in two of the primary centers providing care and support for autistic children in Jeddah.

Jeddah Autism Center Bagedo and Dr. Erfan Hospital, established in 1981 by Sheikh Ahmed Bagedo and Dr. Muhammad Erfan, has over 42 years of experience in psychiatry and neurological care. It has grown to become one of the most prominent and largest hospitals in Jeddah and the Western Region of Saudi Arabia (Jeddah Autism Center Bagedo and Dr. Irfan Hospital team, 2023). The Jeddah Autism Center for Day Care, established in 1993 by the Al-Faisaliah Women’s Charitable Society, was the first facility of its kind in Saudi Arabia. It is a leading social institution providing top educational, rehabilitative programs, and family support for autistic children (Jeddah Autism Center for Day Care, 2023).

### Study subjects

The study included caregivers of autistic children, selected through simple random sampling during follow-up visits of the above-mentioned settings. Caregivers had to meet specific criteria: aged 18 or older, of both genders, responsible for caring for autistic children for at least 6 months, provided informed consent, and were willing to participate in the study.

### Sample size

The sample size was calculated using Raosoft, Inc., estimating a total of 210 caregivers at a 95% confidence level, 50% response distribution, and ±5% margin of error. The population included 459 caregivers attending Jeddah Autism Center Bagedo, Dr. Irfan Hospital, and Jeddah Autism Center for Day Care (400 and 59 caregivers per month, respectively).

### Sampling technique

A probability sampling approach using simple random sampling was used to minimize selection bias and ensure that all eligible caregivers had an equal chance of being included. Proportional allocation was applied to maintain the representation of participants across centers according to their population sizes, thereby enhancing the representativeness and generalizability of the findings. Caregivers were selected randomly from each center using the proportional allocation method based on the following formula:

$$\frac{\text{Sample size}\times \text{Population size within the stratum}}{\text{Total population}}$$

The sample size is distributed as follows Table 1.

**Table 1:** Distribution of the selected autism centers in Jeddah City.

The Randomly selected autism center	Total number of caregivers’ attendees	A selected sample of caregivers was included in the study
Jeddah Autism Center Bagedo and Dr. Erfan Hospital	400	(400 × 210) ÷ 459 = 183
Jeddah autism center for day care	59	(59 × 210) ÷ 459 = 27
Two centers	459	210 caregivers

### Study tools (data collection instruments)

The following tools were used to collect the required data.

#### Tool I: autism stigma knowledge assessment questionnaire

This questionnaire, originally developed by Alkazam et al., consists of 24 items designed to assess caregivers’ knowledge of ASD. Each correct response is awarded 1 point, whereas incorrect and “do not know” responses receive 0. Total knowledge scores range from 0 to 24 and are classified as poor, fair, or good based on percentage thresholds (Alkazam and Al-Dujaili, 2022). Sociodemographic data were also collected, including information related to both the autistic children and their caregivers, such as gender, age, education level, family structure, and occupation.

#### Tool II: the scale of automaticity and repetition in self-stigmatizing thinking (STARS)

The STARS scale, developed by Alshaigi et al., consists of 25 items rated on a five-point Likert scale. It is designed to assess autism-related stigma among caregivers across two dimensions: self-stigma (first section; 14 items) and enacted stigma (second section; 11 items; Alshaigi et al., 2020). Total and subscale scores were calculated and analyzed as continuous variables, with higher scores indicating greater levels of stigma.

#### Tool III: the autism spectrum disorders caregivers’ attitude scale

This scale was adapted from Alyami et al. (2022) and Abdel-Sattar et al. (2022). It measures caregivers’ attitudes toward ASD using self-reported items rated on a three-point Likert scale (3 = agree to 1 = disagree). Total scores range from 5 to 15, with values below the median indicating a negative attitude and scores at or above the median indicating a positive attitude.

#### Tool IV: the world health organization quality of life brief

The WHOQOL-BREF, developed by the World Health Organization in 1996, assesses individuals’ subjective well-being and overall quality of life using 26 items that span four domains: physical health, psychological well-being, social relationships, and environmental conditions. The instrument generates a quality-of-life profile with separate domain scores, in addition to two standalone items evaluating overall quality of life and general health. Higher scores across the domains reflect better perceived quality of life, with domain means representing overall well-being in each area (World Health Organization, 1996a; World Health Organization, 2023; World Health Organization, 1996b).

### Validity and reliability

To ensure content validity, the tools underwent a back-translation process from English to Arabic, followed by review by experts in nursing education. Reliability was assessed through a pilot study involving 10% of the caregivers (21 participants), which helped determine clarity, completion time, and overall comprehension. These participants were excluded from the final sample. The STARS scale demonstrated a Cronbach’s alpha of 0.777, indicating acceptable internal consistency. The ASD Caregivers Attitude Scale showed reliability coefficients ranging from 0.62 to 0.78. The WHOQOL-BREF exhibited strong reliability, with a Cronbach’s alpha of 0.84.

### Data collection procedure

#### Data collection process

Institutional Review Board approval (SP23J/162/11, IRB/2914/23) was obtained from the King Abdullah International Medical Research Center (KAIMRC). Permission to conduct the study was also secured from the directors of the selected centers in Jeddah, KSA. Informed consent was obtained from each participant after they were provided with detailed information about the study’s purpose and procedures. Data collection was carried out from June to August 2024.

#### Data management and analysis plan

Data were coded and analyzed using SPSS (IBM Corp., Version 25). Descriptive statistics—including frequencies, percentages, means, and standard deviations—were used to summarize the sample characteristics and study variables. Pearson correlation coefficients were computed to examine relationships between numerical variables, with statistical significance set at *p* < 0.05. Regression analysis was performed to identify predictors of quality of life (QoL).

### Ethical considerations

Permission to conduct the study was obtained from the Institutional Review Board and the managers of the participating settings. Participants were informed about the study’s purpose and provided with an informed consent form before enrollment. Confidentiality and anonymity were assured, and participants were informed of their right to withdraw from the study at any time without penalty.

## Results

Table 2 presents the sociodemographic characteristics of ASD caregivers and their children. More than half of the children were male (54.8%), with a mean age of 7.2 ± 4.0 years, and nearly half (50.5%) were between 5 and <10 years old. Approximately 40.5% of families consisted of 4–5 members, and 34.3% of the children were the eldest in their households. Regarding caregivers, the mean age was 36.8 ± 8.8 years, with the largest proportion (41.4%) aged 30 to <40 years. The majority were female (72.9%). Most caregivers held a bachelor’s degree (61%), while a small percentage (5.7%) were illiterate. Slightly more than half of the caregivers were employed mothers (50.48%). All caregivers reported awareness of ASD, with nearly half (47.6%) having gained this knowledge through social media.

**Table 2:** ASD caregivers and their children sociodemographic characteristics.

Sociodemographic characteristics	No. (n.210)	%
Child’s sociodemographic characteristics		
Gender		
Female	95	45.2
Male	115	54.8
Age (Years)		
Less than 1	6	2.9
1 to less than 5	67	31.9
5 to less than 10	106	50.5
10 years and more	31	14.8
Mean ± SD	7.2 ± 4.0	
No. of family members		
2–3 persons	68	32.4
4–5 persons	85	40.5
6 persons	57	27.1
Birth order		
First	72	34.3
Second	38	18.1
Third	43	20.5
Fourth or more	57	27.1
Caregiver’s sociodemographic characteristics		
Age (Years)		
20 to less than 30	41	19.5
30 to less than 40	87	41.4
40 to less than 50	67	31.9
50 and more	15	7.1
Mean ± SD	36.8 ± 8.8	
Gender		
Female	153	72.9
Male	57	27.1
Level of education		
Illiterate	12	5.7
High school	50	23.8
Bachelor’s degree	128	61
Master’s degree	14	6.7
PhD or professional degree	6	2.9
Working condition		
Non-working	104	49.52
Working	106	50.48
Heard about autism spectrum disorder		
Yes	210	100.0
Source of information		
Social media	100	47.6
Doctors/health professionals	39	18.6
Relatives	30	14.3
Books/magazines	21	10.0
School/University	20	9.5

Table 3 presents the mean scores of the STARS scale and its subdimensions among caregivers of children with autism. The findings indicate that the mean total stigma score was 63.8 ± 21.8 out of a maximum possible score of 125, suggesting a moderate level of overall stigma among caregivers.

**Table 3:** ASD caregivers’ scale of automaticity and repetition in self-stigmatizing thinking (STARS) assessment scores mean and SD.

ASD caregivers’ scale of automaticity and repetition in self-stigmatizing thinking (STARS) assessment	Maximum allowed scores	Mean ± SD
Self-stigma related to autism	14*5 (70)	35.5 ± 12.3
Enacted stigma	11*5(55)	28.6 ± 10.9
STARS total scores	25*5(125)	63.8 ± 21.8

At the subscale level, the mean score for self-stigma was 35.5 ± 12.3 (out of 70), while the mean enacted stigma score was 28.6 ± 10.9 (out of 55). When considered relative to their respective maximum scores, both subscales appear to fall within the mid-range, indicating that caregivers experience internalized and perceived stigma from others to comparable extents.

The relatively large standard deviations across total and subscale scores suggest considerable variability in caregivers’ stigma experiences, reflecting differences in perceptions, social environments, and individual coping mechanisms.

These findings highlight that stigma remains a notable concern among caregivers of children with autism. The comparable levels of self and enacted stigma suggest that both internal psychological processes and external societal attitudes contribute to caregivers’ experiences. This underscores the need for multifaceted interventions targeting both caregiver support and community awareness to reduce autism-related stigma.

**Figure 2:**
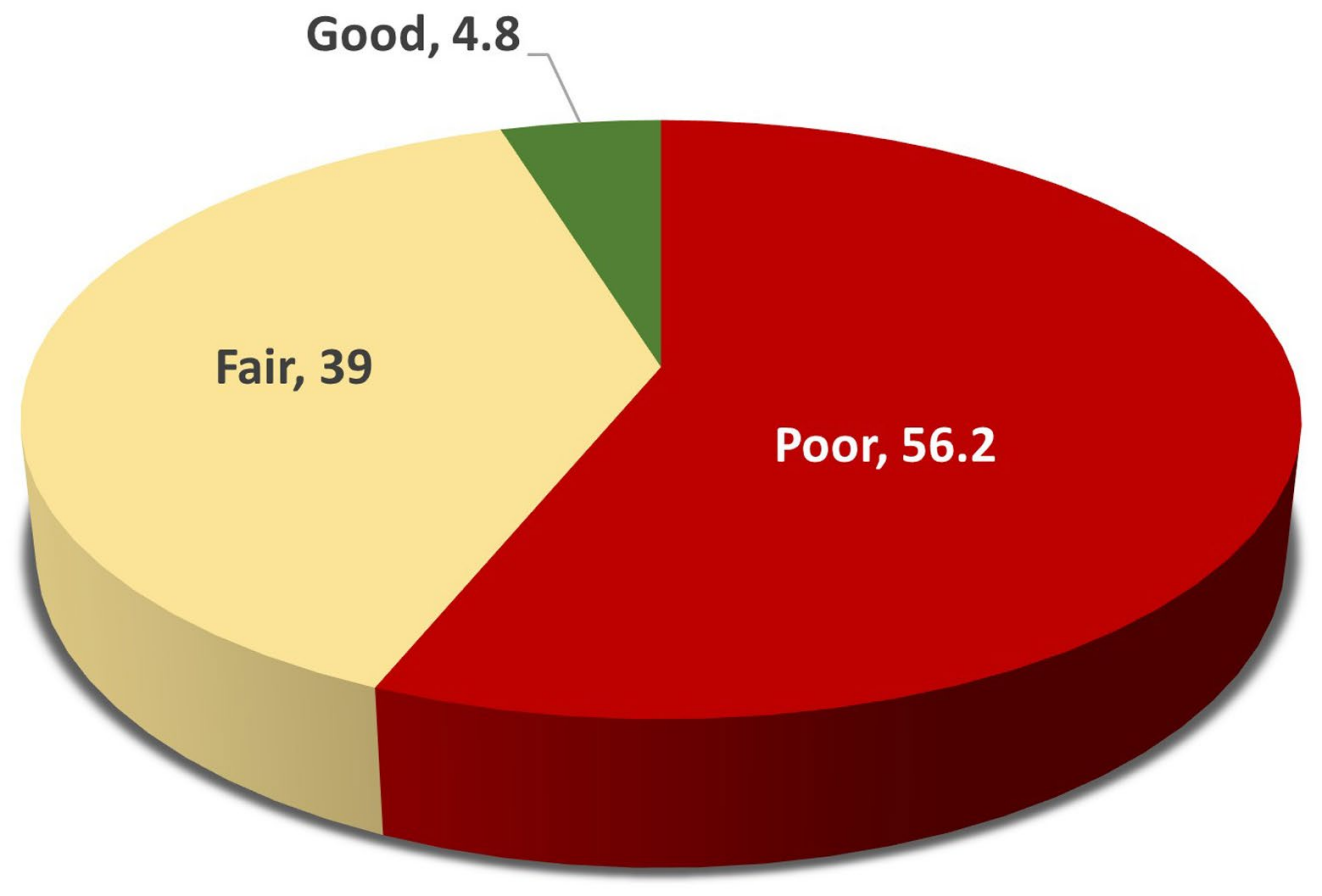
ASD caregivers’ knowledge scores regarding ASD.

Figure 2 illustrates the caregivers’ knowledge scores regarding ASD. More than half of the caregivers (56.2%) demonstrated poor knowledge, 39% had a fair level of knowledge, and only a small proportion (4.8%) achieved a good level of knowledge about autism.

Table 4 presents the attitudes of ASD caregivers toward autism. Nearly 70% of caregivers agreed that it is the parents’ responsibility to seek valuable information about ASD, and slightly less than two-thirds (63.8%) expressed sympathy for individuals with ASD. Half of the caregivers felt equipped to manage children with ASD, while fewer than two-fifths believed that awareness of ASD is sufficient in Saudi Arabia (38.6%) or that autistic individuals can learn without special education (35.2%). Overall, approximately two-thirds of caregivers (62.4%) exhibited a negative attitude toward autism, compared to more than one-third (37.6%) who demonstrated a positive attitude.

**Table 4:** ASD caregivers’ attitude regarding autism.

Items	Agree		Somewhat Agree		Disagree	
	No.	%	No.	%	No.	%
1. Parents are responsible for looking for valuable information about ASD.	145	69.0	51	24.3	14	6.7
2. I feel sympathy for people with ASD.	134	63.8	60	28.6	16	7.6
3. I am equipped to handle children with ASD.	105	50.0	76	36.2	29	13.8
4. Awareness of ASD is adequate in Saudi Arabia.	81	38.6	73	34.8	56	26.7
5. Autistic people can learn without the need to receive special education.	74	35.2	79	37.6	57	27.1
ASD caregivers’ attitudes regarding autism	No. (%)					
Negative attitude	131 (62.4%)					
Positive attitude	79 (37.6%)					

Table 5 presents the quality of life (QoL) of ASD caregivers. About one-third of caregivers rated their overall QoL as good and reported satisfaction with their health (35.2 and 39%, respectively). The mean percent score was highest for the social relationships’ domain (78.0 ± 16.1), followed by physical health (69.9 ± 11.9), psychological health (68.3 ± 11.4), and environmental health (68.3 ± 13.0). The overall QoL mean percent score was 71.1 ± 10.3, with more than three-quarters of caregivers (75.7%) classified as having poor QoL.

**Table 5:** ASD caregivers’ QoL using the WHOQOL-BRIEF tool.

WHOQOL-BRIEF items	No.	%
General items		
Quality of life as perceived by the caregivers		
Very poor	7	3.3
Poor	5	2.4
Neither poor nor good	63	30.0
Good	74	35.2
Very good	61	29.0
Health status satisfaction level		
Very dissatisfied	10	4.8
Fairly dissatisfied	23	11.0
Neither satisfied nor dissatisfied	31	14.8
Satisfied	82	39.0
Very satisfied	64	30.5
Social relation domain		
Mean ± SD	78.0 ± 16.1	
Physical health domain		
Mean ± SD	69.9 ± 11.9	
Psychological health domain		
Mean ± SD	68.3 ± 11.4	
Environmental health domain		
Mean ± SD	68.3 ± 13.0	
QoL classification		
Poor QoL (Lower than median percent score)	159	75.7
High QoL (Higher than median percent score)	51	24.3
Total QoL % score Mean ± SD	71.1 ± 10.3	

Table 6 shows the correlations among ASD caregivers’ quality of life, stigma, knowledge, and attitude. A statistically significant moderate negative correlation was observed between total QoL and total stigma score (*r* = −0.425, *p* = 0.000), indicating that higher stigma corresponds to lower QoL. There was a statistically significant weak positive correlation between total QoL and total knowledge score (*r* = 0.206, *p* = 0.003), suggesting that greater awareness about ASD is associated with slightly better QoL. Additionally, knowledge and attitude are weakly positively correlated (*r* = 0.335, *p* = 0.000), implying that caregivers with more knowledge tend to hold more positive attitudes toward ASD. Other correlations were not statistically significant.

**Table 6:** The correlation matrix of the total quality of life (QoL), scale of automaticity and repetition in self-stigmatizing thinking (STARS) total score, total knowledge score, and total attitude score of the studied ASD caregivers.

Variables	QoL		STARS		Knowledge		Attitude	
	*r*	*p*	*r*	*p*	*r*	*p*	*r*	*p*
QoL			−0.425^**^	0.000	0.206^**^	0.003	0.107	0.121
STARS	−0.425^*^	0.000			−0.028	0.689	0.015	0.829
Knowledge	0.206^**^	0.003	−0.028	0.689			0.335^**^	0.000
Attitude	0.107	0.121	0.015	0.829	0.335^**^	0.000		

r, Pearson Correlation. P, *p* value of Pearson Correlation. *Correlation is significant at the 0.05 level (2-tailed). **Correlation is significant at the 0.01 level (2-tailed). NB: *r* < 0.2: no correlation. *r*: 0.2–0.4: weak correlation. *r*: 0.4–0.6: A moderate correlation. *r*: 0.6–0.8: A strong correlation. *r* > 0.8: A perfect correlation. The color shading included in the Table 6 were used solely to improve readability and visual presentation and do not indicate any specific classification or statistical interpretation.

Table 7 presents the results of a multiple linear regression analysis examining predictors of caregivers’ quality of life (QoL). The overall model was statistically significant, *F* = 7.233, *p* < 0.001, explaining approximately 24.7% of the variance in QoL (Adjusted R^2^ = 0.247). Among the included predictors, child’s gender, child’s birth order, caregiver’s knowledge, and caregiver’s autism-related stigma level (STARS score) emerged as significant predictors of QoL. Specifically, having a female child was associated with lower caregiver QoL (*β* = −0.140, *p* = 0.028), while higher birth order was associated with better QoL (*β* = 0.179, *p* = 0.027). In addition, greater caregiver knowledge significantly improved QoL, *β* = 0.253, *p* < 0.001, whereas higher caregiver stigma was strongly associated with poorer QoL, *β* = −0.401, *p* < 0.001, indicating that caregiver stigma was the most influential predictor in the model. Other demographic variables, including the child’s age, number of family members, caregivers’ age, gender, education, occupation, and attitude, did not reach statistical significance (*p* > 0.05), suggesting that their contribution to QoL was minimal after controlling for other factors. Overall, these findings highlight the importance of reducing stigma and enhancing caregivers’ knowledge to improve the quality of life among caregivers of autistic children.

**Table 7:** Summary of multiple linear regression model for caregivers’ quality of life (QoL) score and related predictors using the standard model.

Model	Unstandardized coefficients		Standardized coefficients	T	Sig.	95.0% confidence interval for B	
	B	Std. error	Beta			Lower bound	Upper bound
(Constant)	1.785	0.287		6.219	0.000	1.219	2.351
Child’s age	−0.001	0.007	−0.012	−0.180	0.858	−0.015	0.012
Child’s gender	−0.120	0.055	−0.140	−2.208	0.028*	−0.228	−0.013
Child’s birth order	0.041	0.019	0.179	2.234	0.027*	0.005	0.078
Number of family members	−0.037	0.019	−0.157	−1.965	0.051	−0.074	0.000
Caregiver’s age	−0.001	0.004	−0.027	−0.357	0.721	−0.009	0.006
Caregiver’s gender	0.082	0.066	0.085	1.236	0.218	−0.049	0.212
Caregiver’s education	−0.054	0.037	−0.098	−1.487	0.138	−0.126	0.018
Caregiver’s occupation	0.012	0.013	0.067	0.984	0.326	−0.013	0.037
Caregiver’s knowledge	0.005	0.001	0.253	3.859	0.000*	0.003	0.008
Caregiver’s attitude	0.000	0.003	0.012	0.193	0.847	−0.005	0.006
Caregiver’s STARS	−0.008	0.001	−0.401	−6.399	0.000*	−0.010	−0.005

Dependent variable: Caregiver’s QOL score. Adjusted R^2^: 0.247; F: 7.233; *p: <0.001.

## Discussion

This study included 210 participants, offering a comprehensive overview of the sociodemographic characteristics of caregivers and their autistic children. Nearly two-fifths of caregivers were aged 30–<40 years, and about three-quarters were females, reflecting the dominant role of mothers in caregiving. More than half were working mothers. Over half of the children were male, and nearly half were between 5–<10 years, indicating a focus on young children. These findings align with Alenazi et al., who also reported a predominance of male ASD children and caregivers aged 31–40 years in Saudi Arabia (Alenazi et al., 2020).

Importantly, regression findings in the current study provide deeper insight into these associations. Child gender, child birth order, caregiver knowledge, and caregiver stigma significantly predicted QoL. Having a female child was associated with lower QoL, which can be logically understood from the Saudi culture’s perspective. Also, a higher caregiver knowledge enhanced their QoL, and stigma was the strongest negative predictor. These results strongly support both the Stress-Process Model and stigma theory, confirming that psychosocial stressors and stigmatizing beliefs directly undermine well-being (Pearlin et al., 1981; Link and Phelan, 2013; Salleh et al., 2024). These findings demonstrate that improving knowledge can buffer stress, while stigma exacerbates psychological and emotional burden, consistent with theoretical expectations. Enhancing knowledge, reducing stigma, increasing community support, and improving access to services can meaningfully improve caregiver QoL.

Consistent with previous literature, this study identified considerable levels of stigma among caregivers, where one-third experienced self-stigma, over two-fifths reported enacted stigma, and about one-third expressed general ASD-related stigma. Similar patterns were described by Alshaigi et al. in Saudi Arabia, while Oduyemi et al. documented even higher levels in Nigeria, likely due to cultural and social differences (Alshaigi et al., 2020; Oduyemi et al., 2021). These findings resonate with stigma theory, which posits that labeling, stereotyping, and discrimination collectively heighten caregiver burden, an effect clearly reflected in the current study’s regression results (Link and Phelan, 2013).

Caregiver knowledge plays a significant role in shaping attitudes, advocacy, and the family’s overall quality of life. In this study, a substantial proportion of caregivers demonstrated poor knowledge of ASD, supporting Alyami et al. (2022). Conversely, Kaman et al. in Malaysia reported higher knowledge levels (Kaman et al., 2023). Limited knowledge is often linked to insufficient public awareness, delayed diagnosis, and restricted access to specialized services. AlAlmaei et al. similarly found that better-informed caregivers exhibit more positive attitudes and improved family well-being (AlAlmaei et al., 2023).

Addressing these systemic issues through education, improved screening, reduced stigma, and equitable access to services can enhance caregivers’ understanding and ability to support autistic children. Implementing programs to increase parental knowledge and exploring ways for communities, organizations, and governments to promote early diagnosis and appropriate treatment plans are essential. Additionally, comparative studies are recommended to assess improvements in caregivers’ knowledge after participating in structured education programs.

A willingness to help individuals with autism is closely tied to caregivers’ attitudes, with positive, accepting, and empathetic attitudes contributing to better outcomes for autistic children. In this study, the Attitude Scale was used to assess these traits, revealing that children thrive when caregivers adopt a strength-based, understanding approach to their unique needs.

Caregivers’ attitudes in this study showed both strengths and areas for development. Nearly 70% believed in the importance of seeking ASD information, and slightly less than two-thirds reported sympathy toward autistic individuals. However, only half felt capable of managing ASD behavior, and fewer than two-fifths believed ASD awareness in Saudi Arabia is adequate. These findings explain why a proportion of caregivers still held negative attitudes, similar to Banda (2021) and Sulaimani et al. (2023). Meanwhile, 37.6% showed positive attitudes, aligning with Saad et al., who reported 70% positive attitudes among Egyptian parents (Saad et al., 2020). These differences reinforce the Stress-Process Model, where primary stressors (child symptoms), secondary stressors (parental role strain), and moderators (knowledge, stigma, coping) jointly shape caregiver outcomes (Pearlin et al., 1981).

Quality of life (QoL) is a central concern for ASD caregivers. In this study, many caregivers reported poor QoL, consistent with Alenazi et al., who found inadequate QoL in more than 60% of families (Alenazi et al., 2020). The lowest median scores were in environmental and psychological domains, similar to findings from Renford et al. (2020), and Likhitweerawong et al., who reported that psychological distress, such as frustration, guilt, or chronic stress, has been extensively documented among ASD caregivers (Likhitweerawong et al., 2020). In contrast, the social domain showed the highest mean score, reflecting strong family ties, consistent with regional cultural norms and corroborated by Renford et al. (2020). Previous studies, such as Jain et al., emphasize that greater awareness and access to resources improve environmental QoL (Jain et al., 2019). Furthermore, Alhazmi et al. emphasized that modifiable factors and support can significantly enhance QoL, providing ASD children with environments conducive to developing social and communication skills (Alhazmi et al., 2023).

Nurses play an essential role in supporting families through education, early guidance, facilitating access to resources, and strengthening coping mechanisms. Collaborative, family-centered care can significantly improve outcomes for both caregivers and autistic children (Eben, 2024). Salleh et al., study findings inform healthcare personnel and policymakers about day-to-day parenting realities, and therefore, an effort to coordinate support services across all disciplines could be made to improve outcomes for both parents and children (Salleh et al., 2022). The current study not only provides valuable insights but also serves as a step toward empowering families and improving the long-term well-being of individuals with ASD.

## Limitations of the study

While this study offers valuable insights into the factors influencing caregivers’ quality of life, several limitations should be noted. First, the cross-sectional design limits the ability to infer causal relationships between autism stigma, knowledge, attitude, and quality of life. Second, data were collected from only two centers in Jeddah, which may limit the generalizability of the findings to other regions. Third, the use of self-reported questionnaires may have introduced response bias, and cultural or psychological factors were not explored in depth. Future longitudinal and mixed-method studies are recommended to validate and expand upon these findings.

## Conclusion

The study found that approximately one-third of caregivers experienced self-stigma, while two-fifths reported enacted stigma. More than half of the caregivers demonstrated poor knowledge of autism, highlighting gaps in understanding the condition and its characteristics, which contributed to negative attitudes in two-thirds of the sample. Caregivers also exhibited poor quality of life, with significant correlations observed between stigma, knowledge, attitude, and overall QoL. Four factors were identified as significant predictors of caregivers’ quality of life: the child’s gender, the child’s birth order, caregiver knowledge, and autism-related stigma.

## Recommendations

Based on the findings, several recommendations are proposed:

For Practice: Establish community-based initiatives and multidisciplinary support networks to improve caregivers’ access to information, early detection services, and psychosocial support.

For Future Research: Conduct interventional, longitudinal, and multi-regional studies to explore causal relationships and assess the effectiveness of educational programs on caregivers’ knowledge, attitudes, and quality of life. Further research should aim to enhance societal understanding and acceptance of autism.

### Implications of the study

For Nursing Practice: Enhance public awareness through outreach and education programs to reduce autism-related stigma and support caregivers’ well-being. Integrate caregiver support and counseling services into community and pediatric nursing practice to promote holistic care for children with autism and their families.

For Nursing Education: Develop targeted training programs for parents of children with autism, such as workshops or online courses covering behavior management, communication strategies, autism characteristics, and available resources. Incorporate autism care content into nursing curricula to strengthen students’ competence and foster positive caregiver attitudes.

For Health Policy and Program Planning: Policymakers should design and sustain national and community programs that promote autism awareness, reduce stigma, and support caregivers. Integrating counseling and support services into primary healthcare and fostering collaboration across sectors can enhance early detection, intervention, and societal understanding of autism.

## Data Availability

The datasets presented in this study can be found in online repositories. The names of the repository/repositories and accession number(s) can be found in the article/supplementary material.
